# Excessive miR-152-3p Results in Increased BAFF Expression in SLE B-Cells by Inhibiting the KLF5 Expression

**DOI:** 10.3389/fimmu.2019.01127

**Published:** 2019-05-22

**Authors:** Shuangyan Luo, Shu Ding, Jieyue Liao, Peng Zhang, Yu Liu, Min Zhao, Qianjin Lu

**Affiliations:** ^1^Department of Dermatology, The Second Xiangya Hospital of Central South University, Changsha, China; ^2^Department of Dermatology, The Third Xiangya Hospital of Central South University, Changsha, China

**Keywords:** SLE, B-cells, miR-152-3p, KLF5, BAFF

## Abstract

The increased BAFF expression in B-cells of patients with systemic lupus erythematosus (SLE) is associated with B-cell hyperstimulation and T-cell hyperactivity, but the underlying mechanisms are still unclear. This study aimed to uncover the mechanisms that regulate the BAFF expression in SLE B-cells. The results demonstrated that the expression of miR-152-3p was significantly increased in SLE B-cells compared with normal controls. This study confirmed that Kruppel-like factor 5 (KLF5) was a direct target of miR-152-3p, and it could bind to the promoter region of BAFF and inhibit its expression in B-cells. The upregulation of miRNA-152-3p expression decreased the KLF5 expression and increased the BAFF expression in SLE B-cells. Knockdown of miR-152-3p expression inhibited the self-reactivity of SLE B-cells, thereby reducing the autoantibody production. The increased miR-152-3p expression in SLE B-cells led to an increase in BAFF expression by inhibiting KLF5 expression. These factors caused B-cell self-reactivity and autoantibody production, allowing participation in the disease process of SLE.

## Introduction

Systemic lupus erythematosus (SLE) is a systemic autoimmune disease that manifests multiple B cell abnormalities including amplification of memory B cells and increased levels of autoantibodies. This subsequently affects multiple organs and systems, causing significant morbidity and mortality (1). The B-cells can up-regulate T-cell activity and immune response by acting as antigen-presenting cells or by producing pro-inflammatory factors and co-stimulatory molecules. Recent studies showed that uncontrolled overactivated B-cells play an important role in the pathogenesis of SLE (2–5).

MicroRNAs (miRNAs) are a class of endogenous non-coding small RNAs that down-regulate the expression of target genes at the post-transcriptional level. Recently, it has been reported that miRNAs are involved in the regulation of innate and adaptive immune systems (6–8). Altered expression of miRNAs is closely related to the occurrence of autoimmune diseases, including SLE (9–12), multiple sclerosis (13–15), and rheumatoid arthritis (16–18). According to a previous study, the expression levels of 371 miRNAs in B-cells were altered in patients with active SLE than in healthy controls by high-throughput miRNA microarray. Among the 371 miRNAs, the miR-1246 expression level was reduced significantly in the B-cells of patients with active SLE. Further, activation of B-cells in lupus decreased the miR-1246 expression through AKT-P53 signaling pathway, which in turn enhanced the early B-cell factor 1 expression, thereby promoting further activation of B-cells (4). Moreover, the miRNA microarray results also showed that the miR-152-3p expression level was increased significantly in B-cells of patients with active SLE. However, little is known with regard to the role of miR-152-3p in the function of B-cells and the pathogenesis of autoimmune diseases.

Kruppel-like factor 5 (KLF5) is a zinc finger–containing protein that belongs to the family of Kruppel-like transcription factors. The members of this family are involved in a widespread biological processes, including embryonic development, control of cellular proliferation and differentiation, and stress response (19). KLF5 can function as a transcriptional activator or repressor and a promoter or inhibitor of cell growth and survival depending on the cellular and genetic context in which it operates (20). Noda et al. found that simultaneous downregulation of KLF5 and friend leukemia integration 1 (FLI1) is a molecular hallmark of systemic sclerosis (SSc) dermal fibroblasts, and mice with double heterozygous deficiency of KLF5 and FLI1 spontaneously developed tissue fibrosis, vasculopathy, B-cell activation, and autoantibody production, which are quite similar to those in SSc. KLF5 and FLI1 interacted with CD19 (a dominant signaling component of a multimolecular complex on the surface of mature B-cells) promoter, whereas deficiency of KLF5 and FLI1 in B-cells caused upregulation of CD19 and IL-6 expression (21). This study suggests that KLF5 may be an important inhibitor involved in the regulation of B cell survival, activation and antibody production.

B-cell-activating factor of TNF family (BAFF) is a crucial B-cell survival factor, and is important in the development of autoimmunity, especially in SLE pathogenesis (22, 23). The serum BAFF level has been reported to increase in patients suffering from SLE (24, 25). Overproduction of BAFF in mice led to the release of large number of mature B-cells and antibodies, including autoantibodies, resulting in the development of an autoimmune disease similar to SLE in humans (26). Belimumab, a monoclonal antibody targeting human BAFF, has been approved by the FDA for targeted therapy for SLE. It can reduce the number of abnormal B-cells that contribute to the aggravation of SLE in patients (27, 28). However, the molecular mechanism underlying the increased BAFF expression in SLE remains to be elucidated.

Therefore, this study aimed to demonstrate a new mechanism of SLE: excessive expression of miRNA-152-3p in SLE B cells targeting inhibition of KLF5 expression, resulting in abnormal increase of BAFF expression, which leads to excessive activation of B cells and synthesis of a large number of autoantibodies.

## Materials and Methods

### Participants

A total of 30 female patients with active SLE were recruited from the outpatient clinics in the Second Xiangya Hospital of Central South University. All patients fulfilled at least 4 of the SLE classification criteria of the American College of Rheumatology (29). Relevant clinical and laboratory information regarding the patients are shown in Table 1. The disease activity of lupus was assessed using the SLE Disease Activity Index (30). Also, 30 female healthy controls were recruited from the medical staff at the Second Xiangya Hospital. This study was approved by the Human Ethics Committee of the Second Xiangya Hospital of Central South University, and written informed consent was obtained from all participants.

**Table 1:** Clinical and laboratory characteristics of the patients with SLE in the study.

**Characteristics**	**Characteristics SLE (*n* = 30)**
Sex [male/female (n)]	0/30
Age (years) [median (range)]	33 (17–59)
SLEDAI score [median (range)]	12 (8–24)
Anti-dsDNA (IU/ml) [median (range)]	391.9 (57.9–825)
C3 (g/l) [median (range)]	0.87 (0.38–1.86)
C4 (g/l) [median (range)]	0.09 (0.07–0.18)
Medications (%)	
Prednisolone or methylprednisolone	30
Hydroxychloroquine	25
Cyclosporine-A	5
Mycophenolate mofetil	4

### Isolation, Culture, and Transfection of B-Cells

B-cells were purified from 60 mL of venous peripheral blood using human CD19 beads according to the protocols provided by the manufacturer (Miltenyi, Bergisch Gladbach, Germany), and were cultured in RPMI 1640 medium (Thermo Fisher Scientific, MA, USA). The B-cells were then transfected with plasmid, agomir, or antagomir using human B-cell Nucleofector Kit and Amaxa Nucleofector System (Lonza, MD, USA). Briefly, the B-cells were harvested and resuspended in 100 μL of human B-cell Nucleofector Solution and then mixed with plasmid, agomir, or antagomir. The mixed solution was then electrotransfected using the Nucleofector program U-015 in the Amaxa Nucleofector. The transfected cells were cultured in RPMI 1640 medium and harvested for 48 h.

### RNA Isolation and Real-Time Polymerase Chain Reaction

Total RNA was isolated from B-cells using TRIzol reagent (Thermo Fisher Scientific). Complementary DNAs (cDNAs) were synthesized from 1 μg of total RNA using a miScript II RT (reverse transcription) Kit (Qiagen, CA, USA). DNA was synthesized from cDNA using a miScript SYBR Green PCR (polymerase chain reaction) Kit (Qiagen). Real-time polymerase chain reaction was performed in triplicates using the ABI Prism 7500 (Thermo Fisher Scientific). The target miRNA expression was normalized to RNU6-2, and the mRNA expression was normalized to GAPDH. The fold change was calculated using the formula 2^−ΔΔ*Ct*^, where ΔΔCt = (Ct_targetgene_ – Ct_internalcontrol_)_sample_ – (Ct_targetgene_ – Ct_internalcontrol_)_control_. Primers for miR-152-3p and RNU6-2 were purchased from Qiagen. Primer sequences for mRNA are shown in Table 2.

**Table 2:** Primer sequences for real-time PCR.

	**Forward primer**	**Reverse primer**
KLF5	ACAAATCAGACAGCAGCAATG	GTGACGGGGGAAAGTAAGTG
BAFF	TGGATGACTCCACAGAAAGGGAG	CTCCCTTTCTGTGGAGTCATCCA
GAPDH	AAGAGCTACGAGCTGCCTGAC	ATGGCCCAGCGGATGAG

### Western Blot Analysis

The B-cells were lysed in protein lysis buffer containing a proteinase inhibitor (Thermo Fisher Scientific). Lysates were then centrifuged for 15 min at 14,000*g* at 4°C, and the protein concentration was determined using the Bradford Protein Assay (Thermo Fisher Scientific). Proteins were separated by sodium dodecyl sulfate–polyacrylamide gel electrophoresis using 10% polyacrylamide gels and then transferred onto polyvinylidene difluoride membranes (Millipore, MA, USA). The membranes were blocked with 5% non-fat dry milk in Tris-buffered saline containing 0.1% Tween-20 buffer and immunoblotted with primary antibodies, including anti-KLF5 (Abcam, Cambridge, UK), anti-BAFF (Abcam), and anti-GAPDH (Cell Signaling, BSN, USA). The band intensity was then quantified using Quantity One software (Bio-Rad, CA, USA).

### Chromatin Immunoprecipitation

Chromatin immunoprecipitation (ChIP) analysis was performed according to the instructions provided in the ChIP Assay Kit (Millipore). In brief, the B-cells were fixed for 10 min at room temperature with 1% formaldehyde. Next, the formaldehyde was quenched by adding glycine to a final concentration of 0.125 M. The pellet cells were centrifuged at 1,500 rpm for 5 min, washed twice with 20-mL ice-cold phosphate-buffered saline, and then lysed. The pellet and resuspended lysates were sonicated to reduce the DNA into 500–1,000 base pair (bp) fragments. Then, 2 μg of anti-KLF5, or control rabbit immunoglobulin G (IgG), was added and incubated overnight at 4°C with rotation. All antibodies were obtained from Cell Signaling. The immune complexes were precipitated with protein A agarose beads, washed, and then eluted in 100 μL of TE containing 0.5% SDS and 200 μg/mL proteinase K. Precipitated DNA was further purified with spin columns before amplification of target DNA by real-time PCR. The primers used were as follows: forward 1 (+53 to +72 bp): 5′ ATCGGAGGGTAAATGCCAG3′ and reverse 1 (+214 to +234 bp): 5′ AGAATAAGTGACCACAGGGA3′; forward 2 (−1,171 to −1,191 bp): 5′ AGGGACAGTTAATATCGTCA3′ and reverse 2 (−1,069 to −1,083 bp): 5′ TCCAGACCCAGGCTTC3′; forward 3 (−1,423 to −1,443 bp): 5′ TCTAATGGACTTTAGGGACT3′ and reverse 3 (−1,326 to −1,344 bp): 5′ TTTGTGAGATTTTGGTGC3′; forward 4 (−2,362 to −2,381 bp): 5′GGGTTTACAAACATCCTTC3′ and reverse 4 (−2,262 to −2,284 bp): 5′CTTACCTATAACTCCCACAATA3′.

### Electrophoretic Mobility Shift Assays

The binding reaction was performed according to the procedures described in the previous study (31). Briefly, a DNA probe labeled with biotin containing the binding site was incubated with nucleoprotein. For supershift assays, 2 μg of KLF5 antibody (Abcam) was added to the binding reaction. Moreover, 200-fold molar excess of the cold unlabeled oligonucleotide was used for competition assays. The reactant was subjected to gel electrophoresis on a native polyacrylamide gel and then transferred onto a nylon membrane. The biotin-labeled DNA probe was detected using the streptavidin–horseradish peroxidase conjugate and the chemiluminescent substrate. Two double-stranded DNA probes harboring the two KLF5-binding sites of the BAFF promoter were synthesized with biotin-labeled 3′ ends (binding site 1: 5′-CGAGGGTGAGGCAGGA-3′, binding site 2: 5′-AAAGGGTGGGAGGGGG-3′; probe 1: 5′-ACTTTGCATAAGGAGAGCGAGGGTGAGGCAGGATTTGCAGTCTAGAAGCCTGGGTCTG−3′, probe 2: 5′- TGGACTTTAGGGACTCAGGGGAAAGGGTGGGAGGGGGGTGAGGAATAAAAGACTAAAA-3′).

### T- and B-Cell Co-stimulation Assays

The B-cells were co-cultured with autologous CD4^+^ T-cells at a ratio of 1:4 in 96-well round-bottomed plates 48 h after transfection according to the procedures described in a previous study (32). On day 4, the medium was supplemented, and then the supernatants were collected on day 8 to measure the IgG concentrations.

### Enzyme-Linked Immunosorbent Assay

Anti-dsDNA and IgG concentrations were measured using the enzyme-linked immunosorbent assay (ELISA) Kit (Abcam) according to the manufacturer's protocols. Three replicate wells were quantified for every sample, and all experiments were performed in triplicate. The optical density values were read at 450 nm using ELx800 Absorbance Microplate Reader (BioTek, VT, USA).

### Statistical Analysis

The data were expressed as mean ± standard deviation (SD). Means of 2 continuous normally distributed variables were compared by independent samples Student's *t*- test. Mann-Whitney U test was used, respectively, to compare means of 2 and 3 or more groups of variables not normally distributed. Correlations were analyzed using Pearson's correlation coefficient. Significance was set as *P* ≤ 0.05. All analyses were performed using the SPSS 19.0 software.

## Results

### Increased miR-152-3p Expression in SLE B-Cells

**Figure 1:**
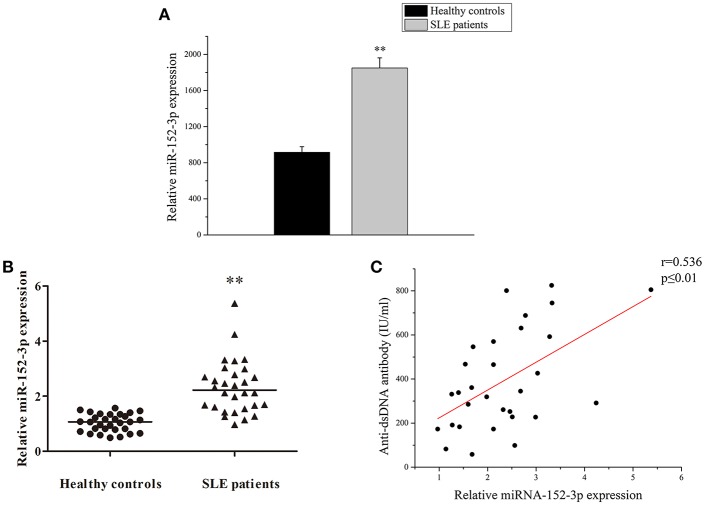
Increased miR-152-3p expression in SLE B-cells. **(A)** The differences in the expression of miRNA-152-3p between SLE and control samples. The variables were compared using the Student *t*-test. **(B)** Relative miRNA-152-3p expression level in B-cells isolated from healthy controls or patients with SLE (*n* = 30 for each group) was assessed by real-time PCR and normalized to RNU6-2. The variables were compared using the Mann-Whitney *U*-test. **(C)** The correlation between miR-152-3p expression and anti-dsDNA antibody level in SLE B-cells (*n* = 30). ^**^P < 0.01.

A high-throughput miRNA microarray of the activities of 371 miRNAs isolated from B-cells of healthy controls and patients with active SLE was conducted in a previous study (4). Among the 371 miRNAs, the miR-152-3p expression level was increased approximately twice in the B-cells of patients with SLE when compared to healthy controls (Figure 1A). In this study, the microarray results from 30 patients with SLE and 30 healthy controls were confirmed by real-time PCR. The results showed that the miR-152-3p expression was significantly increased in patients with SLE when compared with that of healthy controls (Figure 1B). Furthermore, the correlation between miR-152-3p expression and anti-dsDNA antibody was positive (Figure 1C).

### miR-152-3p Promoted B-Cell Responsiveness

**Figure 2:**
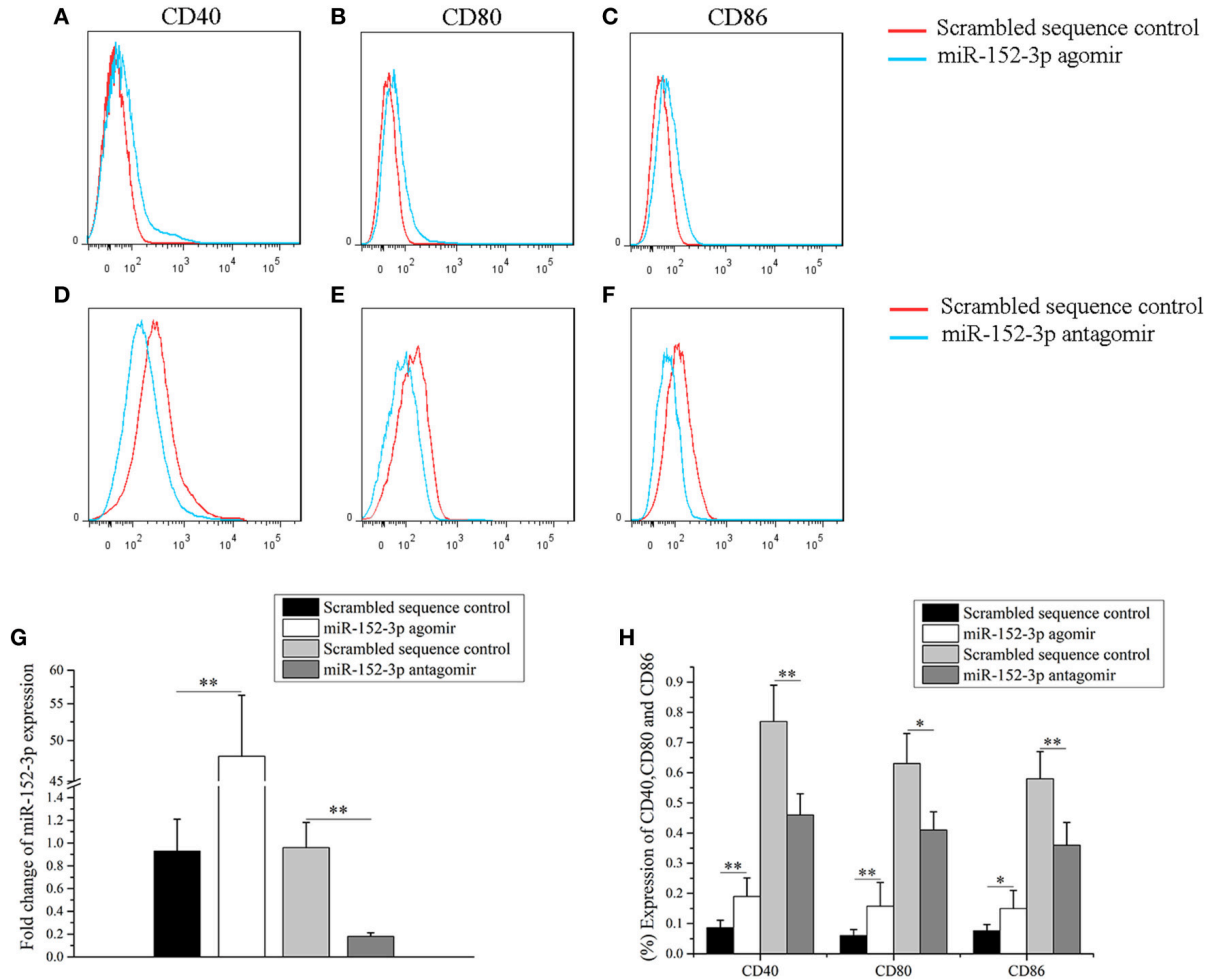
miR-152-3p promoted B-cell responsiveness. **(A–C)** Upregulation of miR-152-3p expression in the B-cells of healthy controls increased CD40, CD80, and CD86 expression levels. Transfected cells were stained with PE-Cy7-conjugated anti-human CD40, FITC-conjugated anti-human CD80, and PerCP-Cy5.5-conjugated anti-human CD86, and then were analyzed by using flow cytometry. **(D–F)** Downregulation of miR-152-3p expression in the B-cells of patients with SLE decreased the expression levels of CD40, CD80, and CD86. Transfected cells were stained with PE-Cy7-conjugated anti-human CD40, FITC-conjugated anti-human CD80, and PerCP-Cy5.5-conjugated anti-human CD86, and analyzed using flow cytometry. **(G)** The expression of miRNA-152-3p in different transfection groups. **(H)** Statistical analysis of percentage of CD40, CD80, and CD86 in different transfection groups. All experiments were performed in triplicate. The variables were compared using the paired *t*-test. ^*^P < 0.05 and ^**^P < 0.01.

To determine whether miR-152-3p upregulation was sufficient to induce responsiveness of B-cells *in vitro*, a miR-152-3p agomir or scrambled sequence control was transfected into B-cells isolated from healthy donors. The cells were collected after 72 h of treatment. Flow cytometric analysis was then performed to determine the expression levels of CD40, CD80, and CD86 in B-cells. Compared with B-cells transfected with scrambled sequence control, there was a significant increase in the expression levels of CD40, CD80, CD86 and miR-152-3p in B-cells transfected with miR-152-3p agomir (Figures 2A–C,G,H). Furthermore, to explore whether miR-152-3p upregulation was necessary for autoimmune reactivity in patients with SLE, primary B-cells from patients with active SLE were transfected with miR-152-3p antagomir or scrambled sequence control. The results showed a significant decrease in the expression levels of CD40, CD80, CD86 and miR-152-3p in SLE B-cells transfected with miR-152-3p antagomir, compared with control-transfected cells (Figures 2D–F,G,H). These results suggested that the upregulated miR-152-3p expression in normal B-cells could promote B-cell hyperresponsiveness.

### Identification of miR-152-3p-Targeting mRNAs in SLE B-Cells

**Figure 3:**
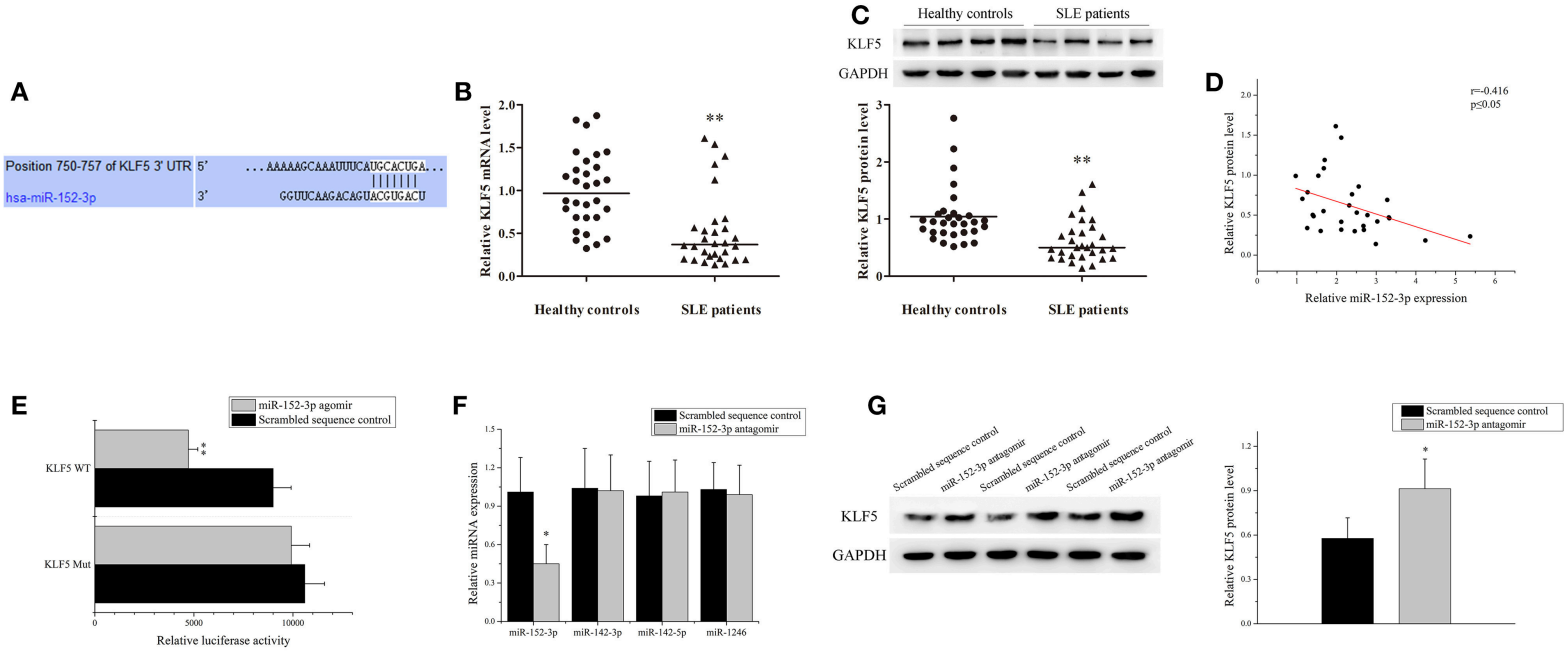
Identification of miR-152-3p-targeting mRNAs in SLE B-cells. **(A)** The sequence of miR-152-3p-binding site in 3′-untranslated region (3′-UTR) of KLF5. **(B)** The KLF5 expression level in B-cells isolated from healthy controls or patients with SLE (*n* = 30 for each group) was analyzed by real-time PCR and normalized to GAPDH. The variables were compared using the Mann-Whitney *U*-test. **(C)** The KLF5 protein expression in B-cells isolated from healthy controls or patients with SLE (*n* = 30 for each group) was analyzed using western blot analysis. One representative blot is shown (upper panel). The intensity of bands was semi-quantitated and normalized to GAPDH (lower panel). The variables were compared using the Mann-Whitney *U*-test. **(D)** The correlation between miR-152-3p expression and KLF5 protein level in SLE B-cells (*n* = 30). **(E)** Relative firefly luciferase activity in Jurkat cells co-transfected with miR-152-3p agomir or scrambled sequence control together with luciferase reporter constructs containing either a WT or a mutated (Mut) KLF5 3′-UTR. Data represents the mean of three independent experiments. **(F)** The miR-152-3p, miR-142-3p, miR-142-5p, and miR-1246 expression levels in SLE B-cells after transfection with miR-152-3p antagomir or scrambled sequence control were analyzed by real-time PCR. All experiments were performed in triplicate. The variables were compared using the paired *t*-test. **(G)** The protein level of KLF5 in SLE B-cells after transfection with miR-152-3p antagomir or scrambled sequence control. All experiments were performed in triplicate. One representative blot is shown (left panel). The intensity of bands was semi-quantitated and normalized to GAPDH (right panel). Data represents the mean of three independent experiments. The variables were compared using the paired *t*-test. ^*^P < 0.05 and ^**^P < 0.01.

According to the TargetScan database (http://www.targetscan.org/vert_71/), KLF5 was found to be a predicted target of miR-152-3p. The position 750-757 of KLF5 3′-untranslated region (3′-UTR) was contained miR-152-3p-binding sites (Figure 3A). The KLF5 expression level in B-cells of 30 patients with SLE and 30 healthy controls was measured. Real-time PCR and western blot analysis results showed that the KLF5 expression level was significantly decreased in patients with SLE when compared with healthy controls (Figures 3B,C). A correlation analysis showed that the KLF5 expression was inversely related to the miR-152-3p expression in SLE B-cells (Figure 3D).

A luciferase reporter assay was used to confirm that KLF5 is a direct target of miR-152-3p. The firefly luciferase reporter vectors that were fused with downstream to a segment of KLF5 3′-UTR containing wild-type (WT) putative miR-152-3p-binding sequence (KLF5 WT-luciferase) or equivalent segment containing three-point mutations in the miR-152-3p-binding sequence (KLF5 Mut-luciferase) were constructed. The constructs were then co-transfected into Jurkat cells together with miR-152-3p agomir or scrambled sequence control. The KLF5 WT-luciferase activity was significantly reduced in cells co-transfected with miR-152-3p agomir when compared with other groups (Figure 3E). Moreover, the B-cells from patients with SLE were transfected with miR-152-3p antagomir or scrambled sequence control. The results of real-time PCR showed that the miR-152-3p expression level was reduced, while the expression levels of unrelated miR-142-3p, miR-142-5p, and miR-1246 remained unchanged (Figure 3F). The protein expression level of KLF5 was significantly increased in miR-152-3p antagomir group when compared with scrambled sequence control group (Figure 3G). Collectively, these data provided evidences that upregulation of miR-152-3p led to decreased KLF5 expression level in SLE B-cells.

### KLF5 Bound to BAFF Promoter and Regulated Its Expression in B-Cells

**Figure 4:**
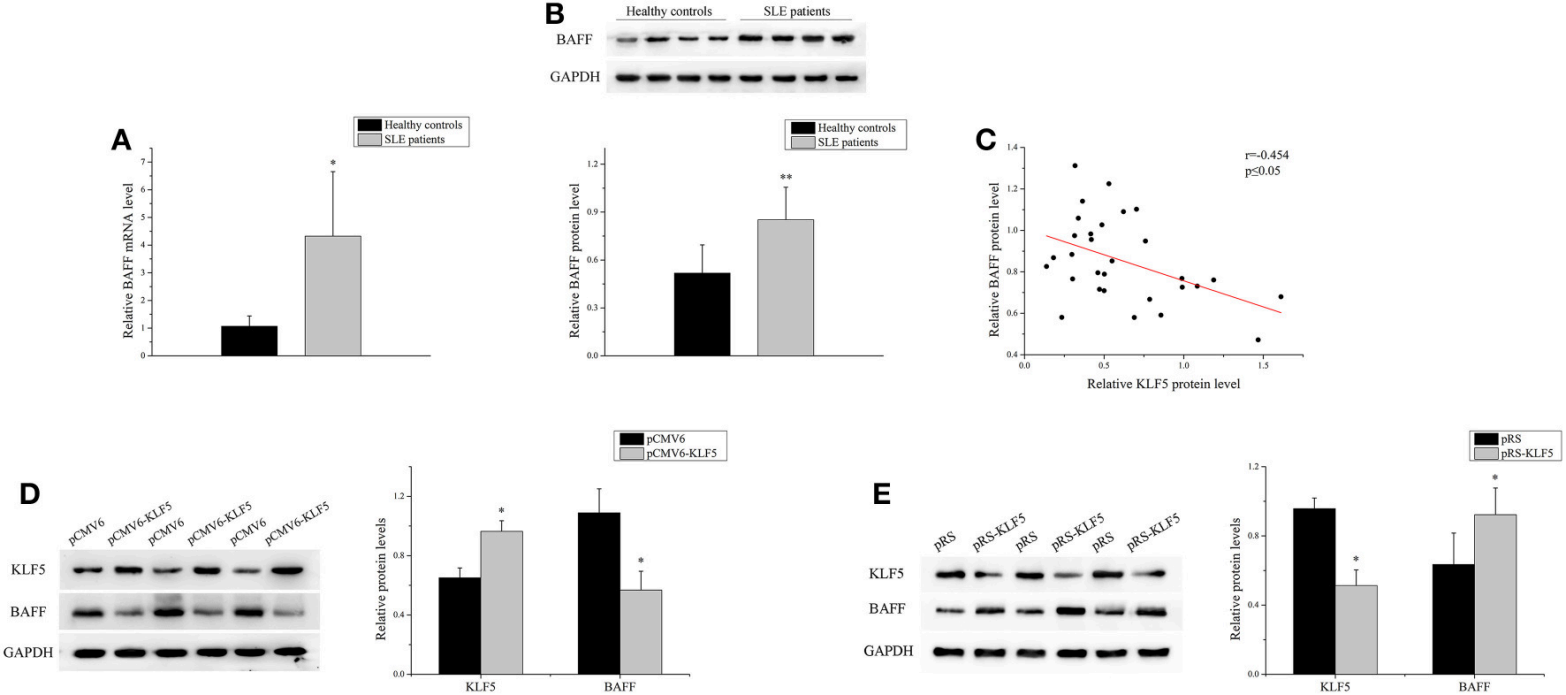
KLF5 regulated BAFF expression in B-cells. **(A)** The relative BAFF level in B-cells isolated from healthy controls or patients with SLE (*n* = 30 for each group) was assessed by real-time PCR and normalized to GAPDH. The variables were compared using the Student *t*-test. **(B)** The BAFF protein expression in B-cells isolated from healthy controls or patients with SLE (*n* = 30 for each group) was analyzed by western blot analysis. One representative blot is shown (upper panel). The intensity of bands was semi-quantitated and normalized to GAPDH (lower panel). The variables were compared using the Student *t*-test. **(C)** The correlation between KLF5 and BAFF protein levels in SLE B-cells (*n* = 30). **(D)** The KLF5 and BAFF protein levels in SLE B-cells after transfection with pCMV6 or pCMV6-KLF5. All experiments were performed in triplicate. One representative blot is shown (left panel). The intensity of bands was semi-quantitated and normalized to GAPDH (right panel). The variables were compared using the paired *t*-test. **(E)** The KLF5 and BAFF protein levels in B-cells of healthy controls after transfection with pRS or pRS-KLF5. All experiments were performed in triplicate. One representative blot is shown (left panel). The intensity of the bands was semi-quantitated and normalized to GAPDH (right panel). The variables were compared using the paired *t*-test. ^*^P < 0.05 and ^**^P < 0.01.

BAFF is necessary for B-cell maturation and survival. In this study, the expression level of BAFF was analyzed in the B-cells of patients with SLE and healthy controls. As shown in Figures 4A,B, real-time PCR and western blot analysis demonstrated that the expression of BAFF was upregulated significantly in SLE B-cells when compared with healthy controls. Correlation analysis showed that the KLF5 expression was inversely related to BAFF expression in SLE B-cells (Figure 4C). Furthermore, the KLF5 expression plasmid pCMV6-KLF5 or negative control was transfected into SLE B-cells, and KLF5 interference plasmid pRS-KLF5 or negative control into normal B-cells. The results of western blot analysis showed that the BAFF expression was downregulated significantly in SLE B-cells with KLF5 overexpression when compared with negative control (Figure 4D). Also the expression of BAFF was upregulated significantly in normal B-cells with KLF5 interference when compared with negative control (Figure 4E).

**Figure 5:**
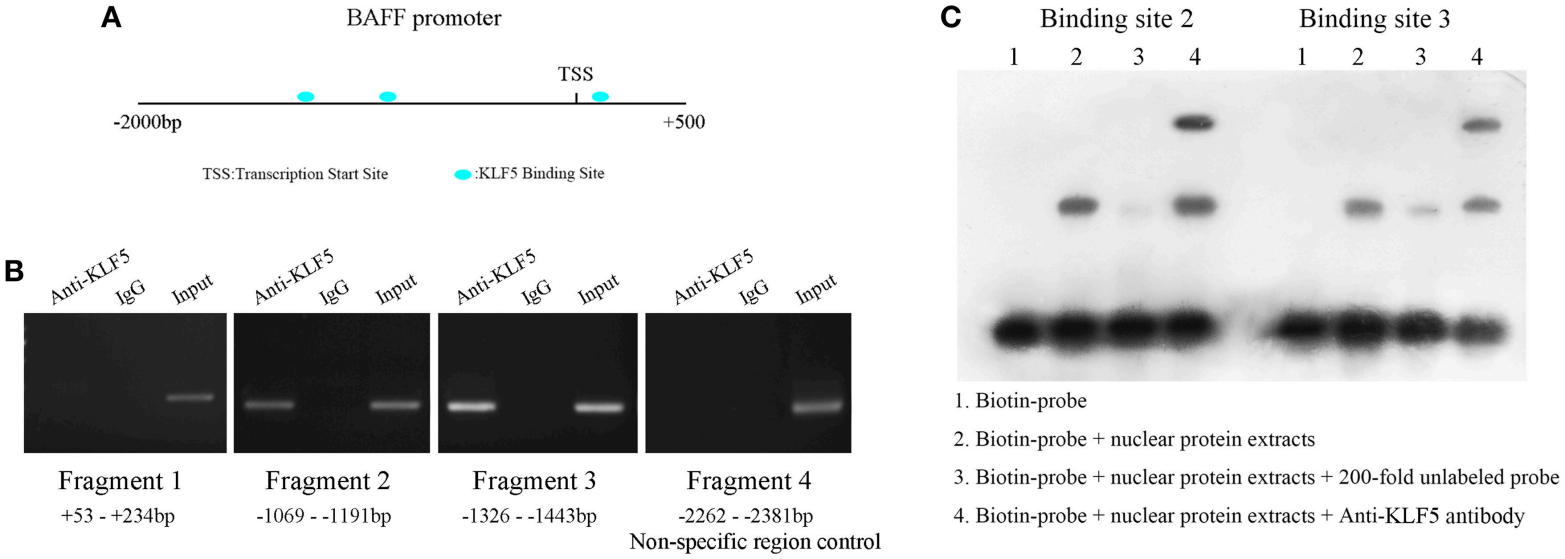
KLF5 is bound to BAFF promoter. **(A)** Prediction of transcription factor binding sites in BAFF promoter region. **(B)** ChIP-PCR showed that KLF5 bound to the promoter of BAFF in B-cells. **(C)** EMSA was used to detect the binding of KLF5 with binding sites 2 and 3 in BAFF promoter region *in vitro*. The biotin probe specific for sites 2 or 3 was bound by nuclear protein extracts of B-cells of healthy controls, which could be blocked by a KLF5 antibody.

Next, to demonstrate the molecular mechanism of BAFF overexpression in SLE B-cells, the JASPAR database (http://jaspar.genereg.net/) was used to predict the transcription factors that might bind to BAFF promoter region. Three binding sites for KLF5 were found at +146 to +162 bp (site 1), −1,094 to −1,110 bp (site 2), and −1,401 to −1,417 bp (site 3) regions upstream of the transcription start site (TSS) of BAFF (Figure 5A). To confirm whether KLF5 can directly bind to BAFF promoter, a ChIP-PCR analysis was performed in B-cells of healthy controls using anti-KLF5 antibody. Four pairs of primers that covered the BAFF promoter +234 to −2,381 bp region were observed (the fourth pair of primer was a non-specific region control). The results showed that KLF5 was bound in the −1,069 to −1,443 bp region upstream of the TSS of BAFF in B-cells (Figure 5B). Moreover, biotin-tagged specific probes were used for −1,069 to −1,127 bp (containing site 2) and −1,380 to −1,438 bp (containing site 3) regions to perform an EMSA with a KLF5-specific antibody. The results of EMSA further validated the conclusion (Figure 5C). Taken together, these results suggested that patients with SLE were manifested with increased BAFF expression in B-cells when compared with that in healthy controls, and the defective expression of KLF5 may be an important reason.

### Knockdown of miR-152-3p Expression Alleviated BAFF Expression in SLE B-Cells

**Figure 6:**
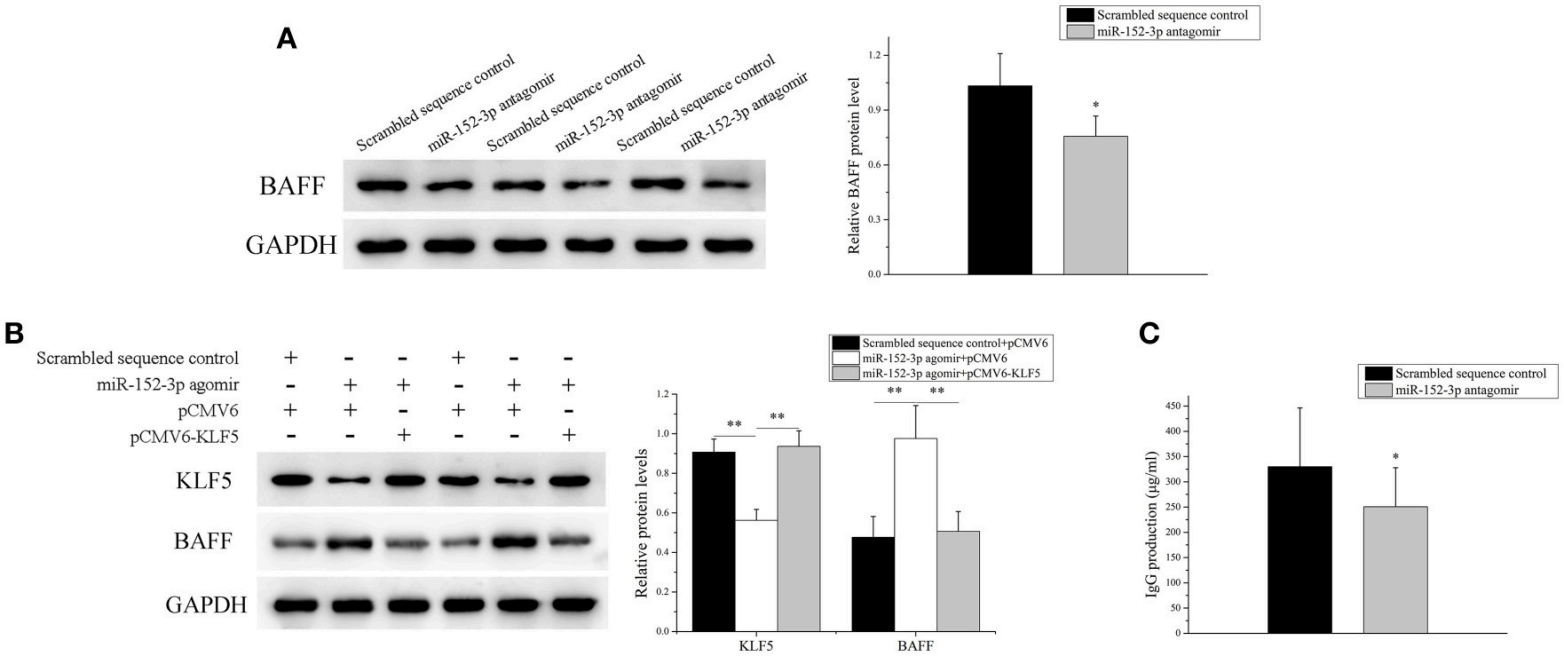
Knockdown of miR-152-3p expression alleviated the BAFF expression in SLE B-cells. **(A)** The BAFF protein level in SLE B-cells transfected with miR-152-3p antagomir or scrambled sequence control was assessed by Western blot analysis. One representative blot is shown (left panel). The intensity of the bands was semi-quantitated and normalized to GAPDH (right panel). All experiments were performed in triplicate. The variables were compared using the paired *t*-test. **(B)** KLF5 and BAFF protein levels in healthy control B-cells after miR-152-3p agomir and pCMV6 or pCMV6-KLF5 co-transfection. One representative blot is shown (left panel). The intensity of the bands was semi-quantitated and normalized to GAPDH (right panel). All experiments were performed in triplicate. The variables were compared using the ANOVA test **(C)** SLE B-cells transfected with miR-152-3p antagomir or scrambled sequence control, where the transfected cells were co-cultured with purified autologous CD4^+^ T-cells. The IgG levels in the culture supernatants were measured using ELISA. Data represent the mean of three independent experiments per group. The variables were compared using the paired *t-*test. ^*^P < 0.05 and ^**^P < 0.01.

The miR-152-3p expression was knocked down in SLE B-cells, and the BAFF mRNA and protein expression levels in SLE B-cells were detected. As expected, the BAFF expression level was decreased significantly (Figure 6A). Also, miR-152-3p agomir and pCMV6 or pCMV6-KLF5 were co-transfected into B-cells isolated from healthy donors. The KLF5 expression was reduced and the BAFF expression level was increased significantly in cells co-transfected with miR-152-3p agomir and pCMV6 when compared with controls (Figure 6B). The KLF5 and BAFF expression levels showed no significant changes in cells co-transfected with miR-152-3p agomir and pCMV6-KLF5 when compared with controls (Figure 6B). To determine the effect of downregulation of miR-152-3p expression on the function of SLE B-cells, SLE B-cells with miR-152-3p interference were co-cultured with purified autologous CD4^+^ T-cells. The IgG levels in the culture supernatants were measured by ELISA. As shown in Figure 6C, the IgG antibody production was significantly lower in miR-152-3p interference group than in negative control group. Together, these results suggested that the regulation of BAFF expression by miR-152-3p depended on KLF5. Knockdown of miR-152-3p expression inhibited the self-reactivity of SLE B-cells, reducing the autoantibody production.

## Discussion

Accumulated evidences showed that miRNAs are important in the pathogenesis of SLE. miR-126 and miR-148a directly inhibited the expression of DNA methyltransferase 1 (DNMT1), and were overexpressed in SLE CD4^+^ T-cells, which thus led to DNA hypomethylation, overexpression of CD11a and CD70, and promotion of CD4^+^ T-cell autoreactivity in SLE (33, 34). Moreover, the overexpression of miR-29b inhibited DNMT1 expression by targeting SP1 in SLE CD4^+^ T-cells (35). In SLE, miR-31 (11), miR-142-3p, and miR-142-5p (10) expression levels were decreased in CD4^+^ T-cells, contributing to dysregulated function of CD4^+^ T-cells and cytokine production. However, these studies mainly focused on T-cells. Few studies have reported on the abnormal expression of miRNAs in SLE B-cells. The miR-30a expression was upregulated in SLE B-cells, which accelerated B-cell proliferation and increased IgG production by targeting mRNA of Lyn, which is a key negative regulator of B-cell activation (36). Cyclin D3 (CCND3) was important in B-cell proliferation, development, and differentiation. The activation of TLR7 increased CCND3 expression by downregulating miR-15b in SLE B-cells (37). Also, miR-152 was overexpressed in the peripheral blood mononuclear cells of patients with SLE (38). In the present study, the miR-152-3p expression was significantly elevated in the activated SLE B-cells when compared with healthy controls. The upregulation of miR-152-3p expression in normal human B-cells could promote the expression of B-cell activation–related genes, CD40, CD80, and CD86. Conversely, the interference of miR-152-3p expression in SLE B-cells could effectively inhibit the expression levels of CD40, CD80, and CD86. The overexpression of miR-152-3p is likely to be one of the reasons for B-cell hyperresponsiveness and SLE induction.

At present, research on KLF5 is mainly concentrated in the field of cancer. In pancreatic, bladder, intestine, colon, and breast cancers, KLF5 enhances cell proliferation, survival, and invasiveness, which is deemed to be an oncogene. In contrast, in esophageal squamous cell cancer, prostate cancer, and acute myeloid leukemia, KLF5 promotes cell differentiation and inhibits cell proliferation, acting as a tumor suppressor (39, 40). Few studies have reported on the relationship between KLF5 and autoimmune diseases. Our study confirmed that KLF5 was the target gene of miR-152-3p. The upregulated miR-152-3p inhibited KLF5 expression in SLE B-cells, resulting in lower levels of KLF5 in B-cells of patients with SLE than in normal controls. Furthermore, KLF5 could bind directly to the BAFF promoter region and inhibit its expression. The inhibition of miR-152-3p expression in SLE B-cells effectively increased KLF5 expression and then reduced BAFF expression, inhibiting self-reactivity and autoantibody production of SLE B-cells. These findings suggest that the lack of KLF5 in B cells may be closely related to the occurrence of SLE.

In humans, BAFF is produced by macrophages, dendritic cells, neutrophils, and T cells (41–44). The earliest reports showed that BAFF is not produced by B cells (42). However, in recent years, more and more studies have confirmed that B cells can produce large amounts of BAFF under pathological conditions, such as B cell lymphomas and autoimmune diseases (45). B cells from B-cell chronic lymphocytic leukemia (46), non-Hodgkin's lymphoma (47), rheumatoid arthritis (48), systemic lupus erythematosus (49), and primary Sjogren's syndrome (50) express high levels of BAFF, which rescues them from apoptosis in an autocrine loop. In this study, we further confirmed the abnormal expression of BAFF in B cells of SLE patients, which provides a new explanation for the anomalous activation of SLE B cells.

## Conclusions

In summary, this study demonstrated that the increased miR-152-3p expression led to increased BAFF expression by inhibiting KLF5 expression in SLE B-cells. These findings elucidated a new molecular mechanism of KLF5-mediated B-cell inhibition. Moreover, it provided an explanation for the overactivation of SLE B-cells caused by the upregulation of miR-152-3p.

## Ethics Statement

This study was approved by the Hunan Ethics Committee of the Second Xiangya Hospital of Central South University, and written informed consent was obtained from all participants.

## Author Contributions

SL, QL and MZ contributed to the design and planning of the experiments. SL and SD provided samples. SL, SD, JL, PZ, and YL conducted the laboratory experiments. SL and SD contributed to the reporting of findings and writing of the manuscript. All authors critically revised the manuscript and gave final approval to the version to be submitted.

### Conflict of Interest Statement

The authors declare that the research was conducted in the absence of any commercial or financial relationships that could be construed as a potential conflict of interest.
